# Examining Influences of Parenting Styles and Practices on Physical Activity and Sedentary Behaviors in Latino Children in the United States: Integrative Review

**DOI:** 10.2196/publichealth.8159

**Published:** 2018-01-30

**Authors:** Ana Cristina Lindsay, Minerva Wasserman, Mario A Muñoz, Sherrie F Wallington, Mary L Greaney

**Affiliations:** ^1^ Department of Exercise and Health Sciences University of Massachusetts Boston Boston, MA United States; ^2^ Department of Nutrition Harvard TH Chan School of Public Health Boston, MA United States; ^3^ Lombardi Comprehensive Cancer Center Georgetown University Medical Center Georgetown University Washington, DC United States; ^4^ Department of Kinesiology University of Rhode Island Kingston, RI United States

**Keywords:** parenting, styles, practices, physical activity, children, Hispanic, Latino

## Abstract

**Background:**

Research indicates that parents influence their children’s physical activity (PA) and sedentary behaviors (SB) through their parenting styles and practices.

**Objective:**

The objectives of this paper were to evaluate existing research examining the associations between parenting styles, parenting practices, and PA and SB among Latino children aged between 2 and 12 years, highlight limitations of the existing research, and generate suggestions for future research.

**Methods:**

The method of this integrative review was informed by methods developed by Whittemore and Knafl, which allow for the inclusion of qualitative, quantitative, and mixed-methods studies. Using the Preferred Reporting Items for Systematic Reviews Meta-Analyses guidelines, five electronic academic databases (PubMed, SPORTDiscus, PsycINFO, PsycARTICLES, and CINAHL) were searched for peer-reviewed, full-text papers published in English. Of the 641 unique citations identified, 67 full-text papers were retrieved, and 16 were selected for review.

**Results:**

The majority of the 16 reviewed studies were conducted with predominantly Mexican American or Mexican immigrant samples, and only 1 study examined the association between parenting styles and Latino children’s PA and SB. Most (n=15) reviewed studies assessed the influence of parenting practices on children’s PA and SB, and they provide good evidence that parenting practices such as offering verbal encouragement, prompting the child to be physically active, providing logistic support, engaging and being involved in PA, monitoring, and offering reinforcement and rewards encourage, facilitate, or increase children’s PA. The examined studies also provide evidence that parenting practices, such as setting rules and implementing PA restrictions due to safety concerns, weather, and using psychological control discourage, hinder, or decrease children’s PA.

**Conclusions:**

Because this review found a very small number of studies examining the relationship between parenting styles and Latino children’s PA and SB, additional research is needed. Given that the majority of reviewed studies were conducted with predominantly Mexican American or Mexican immigrant samples, additional research examining parenting styles, parenting practices, and PA and SB among multiethnic Latino groups is needed to design interventions tailored to the needs of this ethnically diverse population group.

## Introduction

### Background

Latinos or Hispanics (hereafter referred to as Latinos) are the largest and most rapidly growing population group in the United States [1]. Despite recent declines in the prevalence of childhood obesity in the United States [2,3], evidence indicates that racial/ethnic minority children, including Latinos, remain at increased risk of childhood obesity [2,3]. Therefore, childhood obesity among Latinos is a pressing public health concern because child weight status tracks into adulthood [2,3]. In addition, racial/ethnic minority children are at high risk of physical inactivity and increased levels of sedentary behaviors (SB) (eg, watching TV/videos, playing video games, and being on the computer) [3-5].

Physical activity (PA) is a key component of energy balance, and promoting PA is essential to prevent childhood obesity and to promote children’s health [6,7]. Physically active children have healthier cardiovascular profiles, leaner body frames, and higher peak bone mass compared to physically inactive children [4,6,8,9]. In addition to regulating body weight and improving body composition, PA improves psychological and social well-being [4,6,9,10].

Despite the well-documented benefits of PA, children’s PA levels have declined over the past decades, with most children in the United States not getting the recommended 60 min of PA daily [11,12]. Physical inactivity is even a greater problem among racial/ethnic minority children in the United States, with a greater prevalence of physical inactivity among Hispanic children than non-Hispanic white children [13-15]. Therefore, it is essential to understand the factors that influence Latino children’s PA and sedentary levels to address disparities in PA and obesity rates among this population group.

Parents influence their children’s PA and SB, and existing scientific evidence suggests that one way parents do this is through their parenting style [16,17]. *Parenting style* encompasses the overarching attitudes and behaviors that characterize how a parent interacts with a child across domains of parenting [18-20]. Parenting style includes 2 main dimensions: *demandingness* (also defined as control) and *responsiveness* (also defined as warmth). In total, 4 parenting-style typologies have been described: (1) authoritarian (high demandingness/low responsiveness), (2) authoritative (high demandingness/high responsiveness), (3) permissive (low demandingness/high responsiveness), and (4) uninvolved (low demandingness/low responsiveness) [21,22]. Available evidence examining the association between parenting styles and children’s PA and SB is mixed [22-29]. A recent study conducted in the United Kingdom documented that permissive parenting was associated with higher levels of PA among 10- to 11-year-old children [23]. In contrast, a systematic review of general parenting and overweight- and obesity-related behaviors revealed mixed results for an association between parenting styles and children’s PA [17]. Overall, however, results suggest that children raised in authoritative homes are more physically active and have lower body mass index (BMI) levels than children raised to other parenting styles (authoritarian, permissive/indulgent, uninvolved/neglectful) [17].

In terms of PA and SB, parenting practices are context-specific behaviors that parents use to encourage or facilitate their children’s PA and/or reduce SB, such as providing logistic support for PA, encouraging their children to be physically active (eg, verbal encouragement), restricting or setting limits for SB (eg, limiting screen time), monitoring children’s PA and sedentary time, and modeling PA behaviors [22-24,29,30-32]. Moreover, although parenting styles are independent of parenting practices, evidence suggests that parenting styles may facilitate or hinder positive PA parenting practices [23-27,31,33]. For example, parents who exhibit a more controlling parenting style (authoritarian) are more likely to engage in parenting practices such as setting overzealous or strict boundaries on children’s free outdoor play that hinder their children’s PA [23,24,29].

Prior research indicates that cultural norms and values and social context influence parental attitudes toward child-rearing; therefore, the effects of different parenting styles and parenting practices often vary across ethnic groups [34-42]. Available research on general parenting practices suggests that Latino parents’ parenting styles are usually characterized as showing high levels of parental control [40-43] and sometimes (especially maternal) indulgency [37,43,44]. Moreover, available research on PA parenting styles and practices suggests that Latino parents often employ authoritarian and indulgent/permissive styles, and parenting practices that often reflect high control (eg, strict boundaries on children’s free outdoor play) and permissiveness (eg, lack of setting limits on screen time) [35-40,44,45].

### Objectives

Given the low prevalence rates of PA and high rates of SB among Latino children [46] and the increasing evidence linking parenting styles and practices to children’s PA and SB [17,23,27,31,32], the objectives of this integrative review were to: (1) evaluate existing research examining the associations between parenting styles, parenting practices, and PA and SB in Latino children aged between 2 and 12 years; (2) highlight limitations of existing studies; and (3) generate suggestions for future research.

## Methods

### Design

The method of this integrative review was informed by methods developed by Whittemore and Knafl [47], which allow for the inclusion of qualitative, quantitative, and mixed-methods studies. This review had 3 key steps: (1) conducting a systematic literature search; (2) evaluating retrieved studies using a thematic analysis process—data reduction, data display, and drawing and verifying conclusions; and (3) presenting conclusions. In addition, we used the reporting guidelines of the Preferred Reporting Items for Systematic Reviews and Meta-Analysis (PRISMA) statement [48] to guide the inclusion and exclusion of research papers.

### Search Strategy

We searched 5 databases—PubMed, SPORTDiscus, PsycINFO, PsycARTICLES, and Cumulative Index to Nursing and Allied Health Literature (CINAHL). The search, conducted between August 2016 and September 2017, was limited to full-text, peer-reviewed papers published in English before September 2017. Search terms included: (parent* AND (styles OR practice OR strategy* OR behavior) AND (“physical activity” OR exercise OR sedentary behavior*) AND (children OR pediatric) AND (Hispanic OR Latin* OR Mexican* OR Spanish OR immigrant OR ethnic*).

A team of 2 authors (MW, ACL) independently examined the titles and abstracts of all citations identified and excluded citations when both authors determined that the study did not meet the inclusion criteria. Next, the same 2 authors independently reviewed the full-text papers and the reference lists of the studies that were not excluded based on title and abstracts. In addition, the same 2 authors also searched the reference lists of papers that cited the 16 included papers. A final set of agreed-upon papers was examined to extract the relevant information pertaining to the objectives of this integrative review. The search strategy is illustrated in Multimedia Appendix 1.

### Study Selection

Qualitative and quantitative studies examining the influence of parenting styles and parenting practices on PA and SB of Latino children were eligible for inclusion if they met the following criteria: (1) peer-reviewed, full-text papers published in English before September 2017; (2) parents (18+ years old) of children between 2 and 12 years of age; and (3) at least 50% of the total sample self-identified as Latino. Review and validation studies were excluded.

The PRISMA statement guidelines [48] were used to report the review process (see Multimedia Appendix 1). The initial search identified 730 papers. After removing duplicate studies, 641 papers were assessed based on title and abstract by 2 authors independently. Studies were excluded when both authors determined that the study did not meet the inclusion criteria: 574 citations were excluded, and 67 full-text papers were selected for further assessment. Of the 67 full-text papers, 52 did not meet the inclusion criteria upon full-text review and were excluded for various reasons (sample was not at least 50% Latino or studies included special population groups), yielding 15 eligible papers. In addition, to identify additional potentially eligible studies, 2 authors searched the reference lists of full-text papers (n=15) that satisfied the inclusion criteria. This manual search yielded 1 additional eligible study. In total, 16 papers were selected for final inclusion in this integrative review.

### Data Extraction and Synthesis

The 16 identified eligible studies were analyzed and synthesized using the Matrix Method [49], and 2 authors (ACL, MW) independently read all papers and completed a data extraction form created to gather the following information: (1) authors, (2) study setting, (3) study aim(s), (4) study population, (5) study design, (6) measure(s) of parenting styles or PA parenting practices, (7) measure(s) of child’s PA, and (8) study results. The completed data extraction forms were compared, and discrepancies were discussed and resolved with feedback from a third author (MAM). This review synthesizes the extracted data, examining associations between parenting styles or PA parenting practices (exposures) and children’s PA level or SB (outcomes). Due to the range of study designs, assessment of exposure, and outcomes, conducting a meta-analysis was not appropriate, and results of this review are presented as a narrative summary.

### Quality Assessment of Included Studies

Included studies were evaluated using one of two quality frameworks. Quantitative studies were assessed using the appraisal framework [50] to rate study quality, with the maximum score being 5 (Multimedia Appendix 2). Qualitative studies were assessed using the Critical Appraisal Skills Program [51], a 9-question appraisal tool (Multimedia Appendix 3). Two researchers (ACL, MW) independently assessed the study quality using these checklists and discussed and resolved any uncertainty.

#### Quantitative Studies

A total of 11 quantitative studies [52-62] were identified, out of which only one fulfilled all of Glaszious’s methodological criteria [50]. This review found that selection and measurement bias could have possibly affected the results of the other 10 studies; in 5 studies, it was not clear whether participants were randomly selected, and there was limited or no information on comparison between respondents and non-respondents or information on participation rates (Multimedia Appendix 2).

#### Qualitative Studies

Our review indicated that all of the 5 qualitative papers [63-67] were of good quality. Nevertheless, none of the examined qualitative studies considered the relationship between the researcher and participants or other possible power imbalances that might have influenced the studies’ findings (Multimedia Appendix 3).

## Results

### Search Results

After studies were assessed for eligibility using the inclusion and exclusion criteria previously discussed, 16 original research papers were deemed eligible to be included in this integrative review [52-67]. Qualitative and quantitative studies included in this review explored or assessed associations between the following: (1) parenting styles and children’s PA and/or SB, and (2) PA parenting practices and children’s PA and/or SB.

### Study Characteristics

Multimedia Appendix 4 presents a description of included studies, whereas additional study characteristics are presented in Multimedia Appendix 5. Eligible studies examined parenting styles [48] and a range of PA parenting practices [52-65,67] and children’s PA levels and behaviors using a wide variety of research methods. Of the 16 included studies, 1 focused on parenting styles [48] and 15 on PA parenting practices [52-60,63-65,67]. Of the 16 reviewed studies, 11 employed quantitative methods [52-62] and 5 qualitative research methods [63-67]. Of the 11 quantitative studies, 5 were observational cross-sectional studies [58-62], and 6 used quasi-experimental designs to determine intervention efficacy [52-57].

In total, 4 validated questionnaires—the Parenting Strategies for Eating and Activity Scale (PEAS) [68], the Preschooler Physical Activity Parenting Practices (PPAPP) [49], the Activity-Related Parenting Practices Scale [69], and an adapted version of the Girls Health Enrichment Multi-Site Studies (GEMS) [70]—were used in 9 studies [52-60] to assess PA parenting practices. In addition, 2 studies [61,62] used an observational system called Behaviors of Eating and Activity for Child Health: Evaluation System (BEACHES) [71] to assess, among other factors, parenting practices that correlated with children’s PA.

All 16 studies were conducted in the United States, but in only 5 states (California, Massachusetts, North Carolina, Texas, and Wisconsin). Sample sizes ranged from 33 to 812 participants, and the majority of samples were predominantly Mexican American or Mexican immigrant [52-63,65-67], except for 1 study conducted in Massachusetts, which included a multiethnic sample of Latina mothers [64]. Children included in study samples were aged between 3 and 12 years.

This review is organized into 2 sections: (1) studies that explored or assessed associations between parenting styles and children’s PA levels or behaviors and (2) studies that explored or assessed associations between parenting practices and children’s PA levels and/or behaviors or SB. Findings from the 16 reviewed studies are presented and discussed in multiple sections of the synthesis of findings of this review where applicable.

### Synthesis of Findings

Multimedia Appendix 6 presents a synthesis of the 14 themes identified in the examined studies relating to parenting styles and PA parenting practices of Latino parents. Most themes, except for three related to parenting styles, focused on PA parenting practices and fell into either of 2 categories: (1) factors that either encourage, facilitate, or increase PA (ie, parental engagement and/or involvement, verbal encouragement or motivational support, prompting the child to be physically active, providing logistic or instrumental support, limiting SB, using reinforcement and rewards, monitoring) and (2) factors that discourage, hinder, or decrease PA (ie, rules and restrictions, discipline [eg, making children stay inside as punishment], and psychological control [eg, parental criticism, intimidation and insults, manipulation of child’s behavior to satisfy parents’ needs]) in Latino children.

### Parenting Styles

Only 1 study explored the influence of parenting styles on children’s PA. This qualitative study [66] included 4 focus groups conducted with Mexican American or Mexican immigrant mothers and fathers, and its analysis determined that permissive, authoritative, and authoritarian parenting styles were perceived as being related to children’s PA. Findings suggested that Latina mothers’ statements were more reflective of a permissive approach to their children’s PA, whereas the fathers’ approach used authoritative (ie, setting expectations, but willing to negotiate) or authoritarian (ie, dictating what the children do) parenting styles to promote children’s PA.

### Parenting Practices

Of the 16 included studies, 15 examined the association between PA parenting practices and children’s PA and/or SB. Overall, studies showed that parenting practices such as providing verbal encouragement or offering motivational support for the child’s PA, prompting the child to be physically active, engaging and being involved in PA with the child, providing logistic or instrumental support (eg, transportation, enrolling child in sports class), offering positive reinforcement, monitoring the child’s PA and screen time, and setting limits on screen time, encourage, facilitate, or increase children’s PA, whereas parenting practices such as using psychological control, setting rules and restrictions for PA due to the weather (eg, cold weather) and/or safety concerns (eg, traffic, neighborhood violence), using disciplinary action limiting child’s PA (eg, not allowing the child to play outside because of poor behavior) discourage, hinder, or decrease children’s PA (see Multimedia Appendix 4).

#### Logistic or Instrumental Support

Of the included studies, 5 explored the influence of parental logistic or instrumental support on children’s PA [54-57,63]. A qualitative study [63] using focus groups with parents (mothers and fathers) and children (boys and girls aged 10-12 years) revealed that both parents and children viewed parental logistic support for PA as being an important factor for increasing children’s PA [63].

An intervention study conducted in Texas with parents with low incomes and their 5- to 9-year-old children examined the effect of parental support for children’s active living, including parental support for PA and reducing SB. Parental support was assessed using a scale adapted from the GEMS [55] that assessed parent’s verbal support (eg, “I encourage my child to play outside when the weather is nice”), logistic support (eg, “I take my child to his/her sport practice, dance class, or other PA program”), and instrumental encouragement of PA (eg, “I assign active chores for my child such as vacuuming or doing lawn work”). Analysis determined that parental verbal and logistic support for PA reduced children’s SB. In addition, although girls were less sedentary than boys, girls were less affected by parental support [55].

A randomized controlled trial (RCT) testing the feasibility of Helping Healthy Activity and Nutrition Directions (Helping HAND), a 6-month obesity intervention study in primary care settings with 40 parent-child dyads of 5- to 8-year-old children with BMI in the 85th to 99th percentile (82.5% Hispanic), targeted behavior-specific parenting practices, including PA [57]. PA parenting practices were assessed using the Activity-Related Parenting Practices Scale [69]. The intervention changed parenting practices, including logistic support for PA, but did not increase PA. Nevertheless, the intervention group watched less TV than the control group post intervention (14.9 [standard error 2.3] hour/week vs 23.3 [standard error 2.4] hour/week [*P*<.05]).

A study using baseline data from *Aventuras Para Niño* *s* (“Adventures for Kids”), an RCT of an intervention designed to prevent childhood obesity conducted in 13 elementary schools in southern California with children enrolled in kindergarten to second grade (5-9 years of age), showed that parents of children who were overweight provided less instrumental support for their children to engage in PA than parents of children who were not overweight [54] and that provision of instrumental support was associated with children’s PA [54]. A second longitudinal study using data from *Aventuras Para Niño* *s* examined the efficacy of an intervention aimed at improving several dimensions of parenting related to childhood obesity, including selected PA parenting practices (limit setting, monitoring, discipline, control, reinforcement, and support for PA), showed a positive effect of the intervention on parent logistic support for PA [53]. Finally, a third longitudinal study analyzing data from *Aventuras Para Niños*, determined that an increase of PA as reported by the parent was mediated by increases in parental support of a child’s PA (eg, parents providing transportation, encouragement, or actively participating in PA with child) [56].

#### Monitoring

In total, 3 studies explored the influence of the parental practice of monitoring on children’s PA. Arredondo et al examined survey data from parent-child dyads enrolled in *Aventuras Para Niño* *s* study (see above) and determined that parental monitoring of children’s PA and SB was positively associated with children’s PA [52]. Children’s PA was measured using parental reports of their children’s PA level compared with other children, whereas PA parenting practices were assessed using PEAS [68], a self-administered parental questionnaire consisting of a 26-item scale that includes 5 subscales (limit setting, monitoring, discipline, control, and reinforcement). Similarly, a second study also using data from *Aventuras Para Niño* *s* showed that increased PA and reduced SB was mediated by increases in parental monitoring of children’s PA and SB [56]. Finally, a third study designed to examine the efficacy of *Aventuras Para Niño* *s* showed a positive effect of the intervention on the parenting practice of monitoring a child’s PA [53]. The intervention included monthly home visits by a native Spanish-speaking health worker (aka *promotora*) over a 7-month period, plus monthly mailed newsletters [53]. Parents who received the home visits reported more frequent monitoring of their children’s PA, with parents reporting that the children increased their PA and reduced SB.

#### Parental Engagement and Involvement

In total, 5 studies reported on the influence of parental engagement and involvement on children’s PA [58,59,63,65,66]. A qualitative study [66] conducted in Texas with 66 Mexican American and Mexican immigrant mothers and fathers revealed that mothers viewed themselves as being less likely to engage in PA with their children and to have limited involvement in their children’s PA. Fathers viewed themselves as being more physically active, much more engaged in PA with their children, and much more involved in their children’s PA [66]. Similarly, a qualitative study conducted in Wisconsin with primarily Mexican American mothers and fathers and their 10- to 12-year-old children revealed that parental involvement, as perceived by the children, was an important influence on their PA levels [63]. Likewise, a qualitative study using a nominal group technique (a structured multistep group procedure) with 74 parents or legal guardians of 3- to 5-year-old children revealed that parents believed that parental engagement (eg, participating in PA with child, playing sports with child) was a positive PA parenting practice that influenced children’s PA [65]. This finding was further examined in a cross-sectional study with 240 parents in Texas using the validated PPAPP [58], an instrument that assesses parenting practices that encourage and discourage child PA. Results showed that parental perception of a neighborhood’s physical (eg, traffic hazard, safety including stranger danger) and social (disorder) environment was positively associated with parental engagement for promoting the child’s PA [59].

#### Setting Limits

A total of 2 studies explored the influence of parental limit setting on children’s PA. A qualitative study with parents or legal guardians of 3- to 5-year-old children described earlier [65] revealed that parents perceived that not setting limits (eg, allowing child to “watch a lot of TV and use videogames”) discouraged children from being physically active [65]. A study described earlier used baseline data from *Aventuras Para Niño* *s* [54] and found that parents of overweight children set fewer limits on their child’s activities (eg, TV watching) and that their children watch more TV than children who are not overweight.

#### Reinforcement and Reward

Of the selected studies, 2 explored the influence of parents’ use of reinforcement and rewards on children’s PA [52,63]. A qualitative study with parents and children (boys and girls aged 10-12 years) revealed that the use of positive reward for a child being physically active was identified by mothers, fathers, and children as being an important factor in increasing children’s PA [63].

A quantitative study by Arredondo et al [52] determined that parental reinforcement of their children’s PA, as measured by a parent’s praising of a child being physically active, was positively associated with children’s PA [52]. On the other hand, parental use of screen time (eg, offering TV, video, or video games to children as rewards) as a reward for good behavior was not significantly associated with children’s PA.

#### Verbal Encouragement

A qualitative study [63] with parents (mothers and fathers) and children aged between 10 and 12 years revealed that parents’ offering verbal encouragement (eg, telling the child he/she did a good job) of a child’s PA was identified by both parents and children as an important factor in increasing PA in children.

#### Prompting

Of the quantitative studies, 2 [61,62] used the BEACHES [71] observational system (described earlier) and determined that parental prompting of children to be physically active was associated with increased children’s PA levels at home. Moreover, 1 study [62] showed that most activity prompts came from parents interacting with children when the child was sedentary. Furthermore, findings from this study [62] showed that with increasing age, children seem to rely less on the interpersonal interaction with adults for cues to be active.

#### Rules and Restrictions

Of the identified studies, 5 explored the association between setting rules and restricting PA and children’s PA. A qualitative study conducted in Massachusetts using focus groups and in-depth interviews [64] with multiethnic Latina mothers of preschool-age children (2-5 years) revealed that mothers limit their young children’s PA because of safety concerns (eg, perceived neighborhood violence) and weather-related (eg, the cold weather) reasons negatively impacted their children’s PA. Similarly, a more recent qualitative study [67] found that Latina mothers of preschool-aged children who were farm workers living in North Carolina perceived that their concerns about neighborhood safety constrained their children’s PA.

A quantitative study conducted in California with Mexican American preschool-age children (4-year-olds) and parents using the BEACHES observational system (described earlier) determined that parental attempts to control children’s PA through the enforcement of rules (eg, “staying close to the house”; “don’t play rough games”; “no balls in the house”; “don’t run”) were associated with low levels of observed activity [61].

A quantitative study conducted in Texas with 84 children aged between 3 and 5 years and one of their parents who completed the PPAPP (described earlier) showed that the parenting practice of restricting children’s PA because of safety concerns was negatively associated with preschool children’s PA levels [60].

#### Discipline

A quantitative study by Arredondo et al [52] using data from parent-child (kindergarten to second-grade) dyads enrolled in *Aventuras Para Niño* *s* explored the influence of the parenting practice of using discipline, as measured by a parent’s disciplining of the child for watching TV or videos or playing video/computer games on kindergarten to second-grade children’s PA. Findings revealed that parental use of discipline was not significantly associated with children’s PA [52].

#### Psychological Control

In total, 2 studies explored the influence of parental use of psychological control (eg, parental criticism, intimidation, and insults) on children’s PA. A qualitative study with parents and legal guardians of 3- to 5-year-old children (described earlier) revealed that parents perceived psychological control as a negative parenting practice that discourages children from being physically active [65]. An observational study conducted in Houston, Texas, found that parental use of psychological control (manipulation of the child’s behavior to satisfy parents’ needs) was negatively associated with preschool children’s PA levels [60].

## Discussion

### Summary of Findings

A growing body of research indicates that parenting styles and PA parenting practices influence children’s PA levels and behaviors and SB [72-81]. This integrative review identified and synthesized existing research examining associations between parenting styles and practices and PA and SB in Latino children aged between 2 and 12 years. A total of 16 studies met the inclusion criteria of this integrative review, and this small number of studies indicates the need for additional research in this area.

Across the reviewed studies, one qualitative study conducted with Mexican American and Mexican immigrant parents explored the influence of parenting styles on children’s PA, with results showing that maternal permissive parenting styles were viewed as facilitating children’s increased SB (eg, screen time), and paternal authoritative parenting styles encouraged children’s PA [66]. These findings concur with results of a study [23] conducted with white parents, which found that when compared with authoritative maternal parenting, permissive maternal parenting was associated with a higher risk of children exceeding the American Academy of Pediatrics’ guideline recommending that children’s TV viewing should be limited to 1 to 2 hours of quality programming per day [5]. In contrast, some studies suggest that a permissive parenting style facilitates children’s PA [27] and is associated with higher levels of children’s PA [24,27,32].

Researchers suggest that the differential influence of permissive parenting style on PA and SB are consistent with the idea that these two behaviors are separate and distinct [22-24]. Therefore, key predictors of SB and PA such as parenting styles may differ. Nevertheless, available evidence examining the influence of parenting style on children’s objectively measured PA is conflicting, with some studies showing that parenting style does not predict children’s PA [27,30] and others showing a positive association [23]. Given that only one qualitative study included in this review explored the influence of parenting styles on children’s PA, additional qualitative and quantitative studies are needed in this area. As suggested in a recent review by Patrick et al [22], a better understanding of how parenting styles influence parenting practices related to PA and screen-viewing behaviors may generate important insights to the existing evidence base. In addition, most examined studies focused on PA and less on sedentary time, indicating a need for further studies in this area as well.

Studies included in this review provide good evidence for the influence of several PA parenting practices on Latino children’s PA and SB. Provision of logistic or instrumental support, monitoring, use of reinforcement and reward, and limit setting were the most consistent PA parenting practices positively associated with encouraging, facilitating, or increasing Latino children’s PA and decreasing SB (eg, screen time). It is important to note that all of the 6 intervention studies included in this review showed positive effects of the intervention on these PA parenting practices (ie, logistic or instrumental support, monitoring, use of reinforcement and reward, and limit setting). On the other hand, use of rules and restrictions for safety concerns and use of psychological control were associated with discouraging, hindering, or decreasing Latino children’s PA.

Studies included in this review provide consistent evidence for a positive association between the provision of logistic or instrumental support and children’s PA [54-57,63]. Previous studies indicate that logistic or instrumental support can have an important influence on children’s PA [32,82,83].

Consistent with prior research, 3 studies included in this review documented that parental monitoring of children’s PA was positively associated with children’s PA [52,53,56]. Similarly, previous research suggests that parental monitoring, especially maternal monitoring, of a child’s screen time is associated with lower levels of screen time in children [84,85].

Although setting limits may reduce children's screen time, results from a growing literature show mixed evidence that media-related parenting practices are associated with youth screen viewing. It is possible that the lack of association may be partially due to the variety of measures used [23,25,69,84,86-91]. The two studies included in this review that examined the association between limit setting and children’s PA found that allowing a higher limit for sedentary activities (eg, TV viewing and use of video game) was associated with higher screen time [54,65]. The findings of these studies concur with results of a study by Carlson et al [25], which documented that children whose parents report consistent limits about screen time have a lower prevalence of exceeding recommendations for sedentary time than children whose parents do not or are inconsistent in setting screen-time limits [25]. Similarly, in a recent systematic review of screen-time viewing, Hoyos et al [72] found that young children living with less parental screen rules and limit setting were more likely to have higher levels of screen viewing. Likewise, a recent multicenter, cluster RCT conducted in 5 countries (Belgium, Germany, Greece, Hungary, and Norway) with school-age children (10-12 years) found that the presence of rules limiting screen time was significantly associated with less time watching TV/DVD and use of computer/game console time [88]. In contrast, a recent cross-sectional study conducted in the United Kingdom with parents of 6- to 8-year-old children documented that limit setting is associated with greater screen viewing [89]. Given the mixed evidence from existing literature and the paucity of studies among Latino children, further research is needed to examine the associations between limit setting and PA and screen time among Latino children.

Evidence from the extant literature underscores the importance of parental engagement and involvement in promoting children’s PA [6,8,15,17,22,24,29,30,32,33,74,82-84]. Previous studies suggest that parental engagement and involvement, such as playing sports or engaging in PA with children, are positively associated with children’s PA [23,74,85,92]. Studies included in this review concur with these findings [58,59,63,65,66].

Parent’s positive reinforcement of children’s PA has been found to be associated with children’s PA [17,29]. Two studies included in this review showed that use of positive reinforcement (as measured by a parent’s praising of the child being physically active) was positively associated with children’s PA [52,63]. However, parental use of screen time as a reward (eg, offering TV, video, or video games to a child as a reward for good behavior) was not associated with children’s PA [52].

Parental use of verbal encouragement of PA was explored in one qualitative study [63] included in this review. Study findings were consistent with prior studies showing that parent-reported parental verbal encouragement of PA positively influences child-reported PA and discourages time spent watching TV or playing video games [88], and encourages children’s PA behaviors [93,94].

Consistent with research conducted with white children [95] and African American adolescents [84,96], there was good evidence (2/2 studies) [61,62] that parental prompting for children to be physically active encouraged children’s PA. One of these 3 studies [62], which assessed associations at 4 years of age and then again at the 6.5 years follow-up, found that the association of parental prompting for children to be active was particularly influential when children were younger, suggesting that, as children aged, they seemed to rely less on the interpersonal interaction with adults for cues to be active.

There was good evidence (4/4) that parental use of rules about and restrictions of PA because of safety concerns, traffic, or weather discouraged children’s PA [59,60,64,67]. Prior research shows that parents report that their concerns about safety, including concerns related to neighborhood and community safety (eg, crime, traffic), inhibit their children’s PA [29,96-100].

Of the studies included in this review, 2 [60,65] showed negative influence of parental use of psychological control (parental criticism, intimidation, and insults or manipulation of child behavior to satisfy parents’ needs) on children’s PA. There is limited research examining the influence of psychological control on children’s PA [100,101]. Additional research is needed to further explore the influence of psychological control on children’s PA.

### Limitations and Strengths

Our evaluation of the methodologies of studies included in this integrative review suggests some limitations and, therefore, caution in the interpretation of study findings. More than half (69%; 11/16) of the studies included only or predominantly Mexican American and Mexican immigrant parents and children [52-54,59-63,65,66]. Additional longitudinal and intervention studies including multiethnic Latino parents and children are necessary to understand the influence of both parenting styles and PA parenting practices on Latino children’s PA. There was limited or no assessment of the different parenting styles and parenting practices on different parent-child dyad genders (ie, father-son, father-daughter, mother-son, and mother-daughter) in the studies reviewed. Future studies should explore any potential differences and implications for design of interventions.

Furthermore, the majority of included quantitative studies (9/11) used an array of self-reported questionnaires alone or in combination with objective assessments (accelerometers) (2/11) or direct observation (BEACHES) of children’s PA levels (2/11), and self-reported PA data are potentially problematic because of possible parents’ misreporting of children’s PA levels. Variability in the assessment of PA parenting practices (eg, PEAS, PPAPP, GEMS, BEACHES) makes it difficult to compare findings across studies, and caution must be taken in drawing conclusive assertions. Another possible limitation of this review is limited generalizability due the fact that the included studies were conducted in only 5 states (California, Massachusetts, North Carolina, Texas, and Wisconsin).

None of the qualitative studies included in this review considered the relationship between the researcher and participants or other possible power imbalances, which might have influenced the studies’ findings [63-67]. Therefore, future qualitative studies should reflect on how these interactions may impact the study’s results. Finally, publication bias also should be considered, as should the fact that this review is limited to full-text studies published in English and may have excluded studies published in other formats and/or languages.

Strengths of this review include the use of systematic criteria (ie, PRISMA) to identify and select studies and quality assessment tools for the critical appraisals of studies.

### Future Directions

Given the increasing evidence of the importance of parenting styles as a mediator of children’s PA, the paucity of research examining the influence of parenting styles of Latino parents on the PA of Latino children is noteworthy, and further research on this topic is warranted. Moreover, studies including multiethnic Latino parents and children are needed, especially because the majority of studies identified and included in this review were conducted with predominantly Mexican American or Mexican immigrant parents and children [52-54,59-63,65,66]. Given that Latinos represent heterogeneous ethnic groups with a great amount of diversity among Latino individuals, it is important to understand the impact that this diversity has on Latino parenting styles and practices. Additional studies with multiethnic Latino parents and children would help elucidate differentials in parenting styles and PA parenting practices that may exist among various Latino groups. Finally, future research may benefit from further examining differentials of parenting styles and PA parenting practices of multiethnic Latino parents and Latino children’s PA according to the gender of parents and children.

### Conclusions

In summary, this review identified very limited research examining the relationship between parenting styles and PA and SB in Latino children. Given evidence suggesting that parenting styles may facilitate or hinder positive PA parenting practices, future research examining factors associated with PA in Latino children may benefit from greater exploration of the influence of parenting styles on Latino children’s PA and SB.

Studies synthesized in this review provide good evidence for the influence of several PA parenting practices, including offering logistic or instrumental support, monitoring, limit setting, and providing reinforcement and rewards, in encouraging, facilitating, or increasing Latino children’s PA, whereas the influences of parenting practices such as rules, restriction, and psychological control discourage, hinder, or decrease children’s PA. Nevertheless, given that the majority of studies reviewed were conducted with predominantly Mexican American or Mexican immigrant parents and children [52-54,59-63,65,66], further understanding of parenting styles and PA parenting practices among other ethnic Latino groups is likely to provide important insights into the development of interventions tailored to the needs of multiethnic Latino groups and aimed at promoting the PA of Latino children.

Given the increased availability and use of the Internet by Latino parents of various socioeconomic and educational levels [102,103], online interventions (eHealth) [104-106] may offer a viable way to provide health information and professional support to Latino parents regarding PA parenting practices that encourage, facilitate, and increase Latino children’s PA and decrease their SB.

## Appendix

Multimedia Appendix 1 PRISMA Flow Diagram.

Multimedia Appendix 2 Quality assessment: quantitative studies.

Multimedia Appendix 3 Quality assessment: qualitative studies.

Multimedia Appendix 4 Description of studies included in integrative review.

Multimedia Appendix 5 Characteristics of studies included in integrative review.

Multimedia Appendix 6 Synthesis of results of studies included in integrative review.

## Abbreviations

BEACHES: Behaviors of Eating and Activity for Child Health: Evaluation System
BMI: body mass index
GEMS: Girls Health Enrichment Multi-Site Studies
PA: physical activity
PEAS: Parenting Strategies for Eating and Activity Scale
PPAPP: Preschooler Physical Activity Parenting Practices
PRISMA: Preferred Reporting Items for Systematic Reviews and Meta-Analysis
RCT: randomized controlled trial
SB: sedentary behavior
