# Supplementary figures and images for: Examining Influences of Parenting Styles and Practices on Physical Activity and Sedentary Behaviors in Latino Children in the United States: Integrative Review
Source: JMIR Public Health Surveill. 2018 Jan 30;4(1):e14. doi: 10.2196/publichealth.8159 (PMC5811651; doi:10.2196/publichealth.8159)

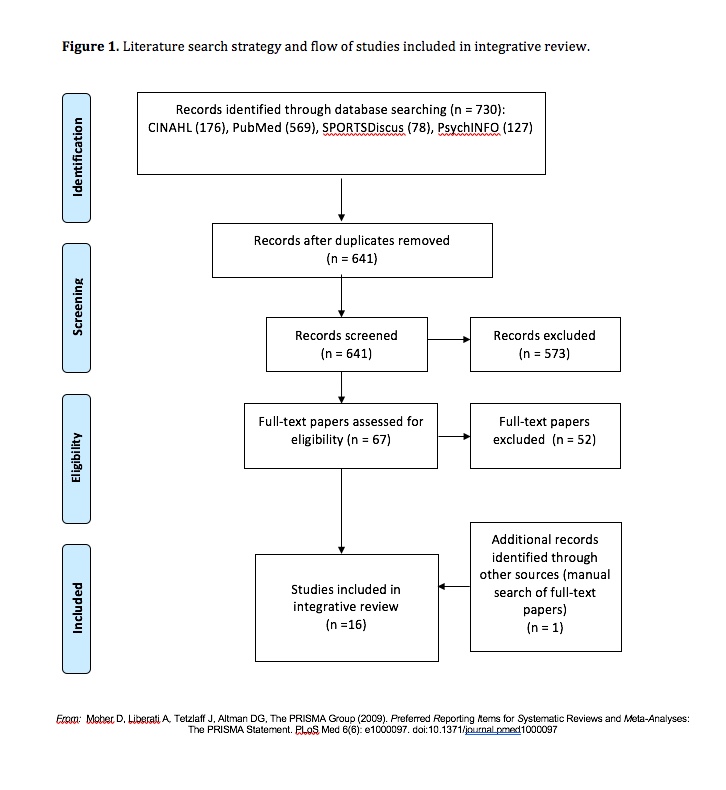

Supplement: Multimedia Appendix 1 [file publichealth_v4i1e14_app1.jpg]
