# Supplementary material for: Examining Influences of Parenting Styles and Practices on Physical Activity and Sedentary Behaviors in Latino Children in the United States: Integrative Review
Source: JMIR Public Health Surveill. 2018 Jan 30;4(1):e14. doi: 10.2196/publichealth.8159 (PMC5811651; doi:10.2196/publichealth.8159)
Supplement: Multimedia Appendix 2 [file publichealth_v4i1e14_app2.pdf]

Mutimedia Appendix 2. Quality criteria quantitative studies adapted from [38].

**Quality Criteria: Quantitative Studies**						
**Studies**	**I. Minimizing selection bias:**				**II. Minimizing measurement bias:**	
	1. Were study participants well defined (time, place, and personal characteristics for eligibility criteria)?	2. Was sample selection random?	3. Was participation rate ≥60%	**OR** If participation rate is <60%, is there a comparison of respondents/non-respondents?	4. Did study use a validated questionnaire?	5. Did the paper report ethical review?
**Sallis et al. 1993**	Y	N	Y	Y	Y	Y
**Elder et al. 1998**	Y	U	U	_	Y	Y
**Arredondo et al. 2006**	Y	N	U	_	N	Y
**Ayala et al. 2010**	Y	N	U	N	Y	Y
**Elder et al. 2010**	Y	N	U	_	N	Y
**Cong et al. 2011**	Y	U	U	N	U	Y
**O'Connor et al. 2013**	Y	N	U	U	Y	Y
**Crespo et al. 2012**	Y	N	Y	_	Y	Y
**O'Connor et al. 2014a**	Y	Y	Y	Y	Y	Y
**O'Connor et al. 2014b**	Y	Y	U	U	Y	Y
**Cerin et al. 2016**	Y	Y	U	U	Y	Y

Y=yes, N=no, U=uncertain.
