# Supplementary material for: Examining Influences of Parenting Styles and Practices on Physical Activity and Sedentary Behaviors in Latino Children in the United States: Integrative Review
Source: JMIR Public Health Surveill. 2018 Jan 30;4(1):e14. doi: 10.2196/publichealth.8159 (PMC5811651; doi:10.2196/publichealth.8159)
Supplement: Multimedia Appendix 3 [file publichealth_v4i1e14_app3.pdf]

Mutimedia Appendix 3: Methodological assessment of qualitative studies [39].

| **Qualitative Evaluation Criteria** | **Snethen et al. 2007** | **Lindsay et al. 2009[a]** | **O'Connor et al. 2013** | **Turner et al. 2014** | **Grzywacz et al. 2016** |
|---|---|---|---|---|---|
| Was there a clear statement of the aims of the research? | Y | Y | Y | Y | Y |
| Was the research design appropriate to address the aims of the research? | Y | Y | Y | Y | Y |
| Was the recruitment strategy appropriate to the aims of the research? (e.g., How were participants selected? e.g. purposive, convenience, consecutive, snowball?; How were participants approached? e.g. face-to-face, telephone, mail, email?) | Y | Y | Y | Y | Y |
| Were data collected in a way appropriate to address the research questions? (e.g., Were questions, prompts, guides provided by the authors? Was it pilot tested? Was data saturation discussed?) | Y | Y | Y | Y | Y |
| Has the relationship between researcher and participants been considered? | N | N | N | N | N |
| Have ethical issues been taken into consideration? | Y | Y | Y | Y | Y |
| Was the data analysis sufficiently rigorous? | U | Y | Y | Y | Y |
| Is there a clear statement of findings? | Y | Y | Y | Y | Y |
| Is the research valuable? | Y | Y | Y | Y | Y |

Y=yes, N=no, U=uncertain.

[*a] Study conducted by the same primary author of this review.
