# Supplementary material for: Examining Influences of Parenting Styles and Practices on Physical Activity and Sedentary Behaviors in Latino Children in the United States: Integrative Review
Source: JMIR Public Health Surveill. 2018 Jan 30;4(1):e14. doi: 10.2196/publichealth.8159 (PMC5811651; doi:10.2196/publichealth.8159)
Supplement: Multimedia Appendix 4 [file publichealth_v4i1e14_app4.pdf]

Mutimedia Appendix 4: Description of studies included in integrative review of parenting styles and practices as mediators of physical activity in Latino children.

**Characteristics**	**No. of Studies**
**Total number of studies selected**	16
**Publication dates**	
Before 2000	2
2000–2005	0
2006–2011	6
2012–2017	8
**Study design**	
Qualitative	5
Cross-sectional	5
Randomized trial/intervention	6
**Age group**	
2–5 years	7
4–12 years	9
**Description of Hispanic/Latino subgroups**	
Multiethnic, description of Hispanic subgroups	3
Multiethnic, no description of Hispanic subgroups	1
Hispanic/Latino only, no subgroup description	7
Hispanic/Latino only, with subgroup description	5
