# Supplementary material for: Examining Influences of Parenting Styles and Practices on Physical Activity and Sedentary Behaviors in Latino Children in the United States: Integrative Review
Source: JMIR Public Health Surveill. 2018 Jan 30;4(1):e14. doi: 10.2196/publichealth.8159 (PMC5811651; doi:10.2196/publichealth.8159)
Supplement: Multimedia Appendix 5 [file publichealth_v4i1e14_app5.pdf]

Mutimedia Appendix 5: Characteristics of studies included in the integrative review.

Author	Method	Country (State)	Participants	Primary Data Collection Method
Arredondo et al. 2006 [40]	Quantitative	U.S. (California)	n = 812; parents; Mexican immigrant/ Mexican-American mothers and K-2nd grade children	Self-administered questionnaire
Snethen et al. 2007 [41]	Quantitative	U.S. (Wisconsin)	n = 36; 12 Mexican- American or Mexican-immigrant fathers,12 mothers, and 12 children (8 boys and 4 girls) age	3 focus groups
Lindsay et al. 2009 [42]	Qualitative	U.S. (Massachusetts)	n = 51; multi-ethnic Latina mothers of preschool-age (2-5 years) children	6 focus groups (N = 31); 20 in-depth interviews
Ayala et al. 2010 [43]	Quantitative	U.S. (California)	n = 811; predominantly Mexican immigrant/ Mexican-American mothers and K-2nd grade children	Self-administered questionnaire
Elder et al. 2010 [44]	Quantitative	U.S. (California)	n = 745; predominantly Mexican immigrant/ Mexican-American parents and K-2nd grade children	Self-administered questionnaire
Cong et al. 2012 [45]	Quantitative	U.S. (Texas)	n = 416; Hispanic low-income parents and their 5- to 9-year-old children	Self-administered questionnaire
Crespo et al. 2012 [46]	Quantitative	U.S. (California)	n = 808; predominantly Mexican immigrant/ Mexican-American mothers and K-2nd grade children	Self-administered questionnaire
O'Connor et al. 2013 [47]	Qualitative	U.S. (Texas)	n = 74; Latino parents or legal guardians of children 3-5 years old	10 nominal group technique; a structured multi-step group procedure
O'Connor et al. 2013 [48]	Quantitative	U.S. (Texas)	n = 80; 40 parents and 40 children (82.5% Hispanic) 5- to 8- years old	Interviewer-administered questionnaire
O'Connor et al. 2014 [49]	Quantitative	U.S. (Texas)	n = 240; Latino parents and their 3- to 5-year-old child	Self-administered questionnaire; accelerometer
O'Connor et al. 2014 [50]	Quantitative	U.S. (Texas)	n = 240; Latino parents (68.7% from Mexico) and their 3- to 5-year-old child	Self-administered questionnaire
Turner et al. 2014 [51]	Qualitative	U.S. (Texas)	n = 33; Mexican-American or Mexican immigrant mothers and fathers and their 5- to 12-year-old children	12 focus groups
Cerin et al. 2016 [52]	Quantitative	U.S. (Texas)	n = 84; Latino parents (74% from Mexico) and children 3-5 years	Self-administered questionnaire; accelerometer
Grzywacz et al. 2016 [53]	Qualitative	U.S. (North Carolina)	n = 33; Latino farmworker mothers of preschool-age (2-5 years) children	33 in-depth interviews
Sallis et al. 1993 [54]	Quantitative	U.S. (California)	n = 387; Anglo-American (n=146) and Mexican-American (n=241) parents and their 4 year-old children	Direct observations using the Behaviors of Eating and Physical Activity for Child Health: Evaluation System (BEACHES) and interviewer-administered questionnaire
Elder et al. 1998 [55]	Quantitative	U.S. (California)	n = 291; parents; Anglo-American(n=178) and Mexican-American (n=113) parents and their children 4 years (Measurement Wave 1) and 6.5 years (Measurement Wave 2)	Direct observations using the BEACHES
