# Supplementary material for: Examining Influences of Parenting Styles and Practices on Physical Activity and Sedentary Behaviors in Latino Children in the United States: Integrative Review
Source: JMIR Public Health Surveill. 2018 Jan 30;4(1):e14. doi: 10.2196/publichealth.8159 (PMC5811651; doi:10.2196/publichealth.8159)
Supplement: Multimedia Appendix 6 [file publichealth_v4i1e14_app6.pdf]

Mutimedia Appendix 6: Synthesis of results of studies included in integrative review.

Category	Themes	Factors That Encourage/Facilitate/Increase or Discourage/Hinder/Decrease Children's Physical Activity
**Parenting Styles**		
	Permissive	(–/+) not setting limits (e.g., allowing child unrestricted TV-viewing and/or other screen time)
	Authoritative	(+) setting expectations, but willing to negotiate
	Authoritative	(–) dictating what the child does
**Parenting Practices**		
	Engagement and Involvement	(+) participating in PA with child; being involved in child's activities (e.g., coaching a sport)
	Verbal Encouragement	(+) praising child for being physically active
	Prompting	(+) prompting child to be physically active by providing verbal or nonverbal encouragement for the child to engage in physical activity
	Motivational Support	(+) praising child for participating in PA
	Logistic or Instrumental Support	(+) providing transportation; enrolling child in PA classes and sports; taking child to park, etc.
	Limit Setting	(+) setting limit on screen time
	Monitoring	(+) monitoring screen time use/monitoring physical activity
	Reinforcement and Rewards	(+) providing positive reinforcement or rewarding child for being physically active or for good performance in sports
	Rules and Restrictions	(–) having rules and restricting outdoor play due to safety concerns (neighborhood safety, traffic, weather, etc.) or weather-related reasons
	Discipline	(0) disciplining child for watching TV or videos, playing video games or the computer
	Psychological Control	(–) parental criticism, intimidation, and insults; manipulation of child's behavior to satisfy parents' needs

(+) indicates encourages, facilitates, or increases physical activity; (–) indicates discourages, hinders, or decreases physical activity; (0) indicates no association with children's physical activity.
